# Medical News and Miscellany

**Published:** 1888-12

**Authors:** 


### MEDICAL NEWS AND MISCELLANY.

---

DR. A. C. WALKER, of Rockdale, has gone to New York to attend the Polyclinic.

---

DIED.—Dr. Sylvester L. Bedall, at Eagle Pass, November 18, 1888, of lead poisoning and obstruction of bowels.

---

MARRIED—In Bristol, Tenn., December —, Dr. F. A. Maxwell, of Del Valle, Texas, to Miss Ella King, of Tennessee.

---

DR. P, ORNELAS, of San Antonio, has been summoned to the City of Mexico by telegram, and the *quid nuncs* say he is to be offered a *po? te-folio* in President Diaz's cabinet.

DOCTORS are migratory animals, and many seem to forget the old saying,—"a rolling stone gathers no moss." We call the attention of those who contemplate a move, to several notices of practices for sale in this issue.

---

FOR PROMPTNESS in filling orders, for skillful packing, better and larger plants and bulbs, and for less money than most others, we commend Messrs. Good & Reese, the Champion City nurserymen of Springfield, Ohio. Send for catalogue and mention this JOURNAL.

---

A DOCTOR ASSASSINATED.—Dr. H. M. Biedler, an old and respected physician of Texarkana, was cruelly murdered last week by a boy fourteen years of age, by name of Ed. Spears. He was shot down on the public street, the boy waylaying him with a double barreled gun, loaded with buckshot. The doctor had had a fight with the boy's father, and had given him the worst of it. Young Spears was admitted to $5000 bail.

---

DEATH OF DR. WHITE, of Cuero.—As we go to press we hear the sad tidings of the death of Dr. A. C. White, of Cuero. He died December 5, in the 53rd year of his age. Dr. White was a native of Virginia, a graduate ot the New Orleans Medical College, and a Confederate Surgeon in Sibley's brigade during the war. He was well known as one of the leading physicians of South Texas, and was a valued member of the Texas State Medical Association. He leaves a wife and five children.

"El Paso, as a Health Resort," is the caption of a very able paper by Doctor Wm. M. Yandell of that city, addresssd to a committee of enterprising citizens, and published, we presume, for general distribution.

The doctor, with great care and patience, studied the tables of the signal service, and upon the records, based a scientific argument in favor of El Paso, that must carry conviction to every reader.

We congratulate the people of that favored spot upon their natural advantages, and upon having a health officer who can so clearly demonstrate the fact to the outside world.

---

A Rare Opportunity.—*Important to physicians seeking a change of location*, or *to an invalid physician!* There is offered a delightful home and farm, complete, with a good practice, in a rich agricultural region, thickly peopled with the better class of farmers. It is in a village of two hundred and fifty to three hundred inhabitants, (five hundred in the school district), and lying on the Aransas Pass Railroad (a depot), in Southwest Texas, about sixty miles from San Antonio,—in the world renowned section of country where consumption cannot exist. Right of way to the Guadaloupe river, very near by, for stock; forty acres of land under first-class fence, twenty-five acres in cultivation, rich black sandy loam, very productive, field perfectly level, and not a stump or tree or stone in the enclosure of twenty-five acres. It is valley land shut in by mountain ranges, and almost perpetual summer prevails. The house is nearly new, a frame dwelling of five rooms, beautifully located, with a fifteen acre lawn in front, planted in evergreens; and there is every appurtenance, such as good barn, cistern of excellent water, etc., etc. A snug "doctor's office" is situated in the corner of this lawn, facing the main street. A christian community, with churches and schools, good schools now in operation, no whisky shops, and no loafers. The practice will net $1,000 to $1,500, cash, and much can be collected in the way of cattle and produce easily converted into cash, as there is good market for everything raised in the valley.

*Twenty-five hundred dollars* will buy house and farm, together with kitchen and household furniture, including a good piano, stock and implements, horse and buggy, cow and calf, office furniture—everything as it stands, (except apparel and feather beds,) ready for one to step in and go to hosue-keeping and practicing medicine. Delightful climate, good society, country settled up—dwellings every one-half mile or mile along the roads, roads good except in very heavy rains—being such that a doctor can use his buggy the year round, and seldom have to go horseback. Terms, two-thirds cash, balance in two years, or one-half cash, balance in one year, vendor's lien, and ten per cent. interest.

The place cannot be duplicated for the money, in Texas! Owner settled there five years ago, for wife's health (phthisis). She has recovered, and wants to return to her old home.

This is a rare chance. Such locations are not "picked up" every day. *No competition* in a radius of twenty miles—ten miles each way! Address the EDITOR OF THIS JOURNAL.

[The owner says, "this people deserve a good doctor, and to such I will give a good 'send off.' Just practice enough to keep one interested, and not enough to break him down. Collections a No. 1! and the farm produces a surplus which is easily sold. Good pasturage. Speak quick, for this is indeed a rare opening." —ED.]

